# Handling tRNA introns, archaeal way and eukaryotic way

**DOI:** 10.3389/fgene.2014.00213

**Published:** 2014-07-10

**Authors:** Tohru Yoshihisa

**Affiliations:** Graduate School of Life Science, University of HyogoAko-gun, Hyogo, Japan

**Keywords:** tRNA, intron, splicing, genome, archaea, eukaryote

## Abstract

Introns are found in various tRNA genes in all the three kingdoms of life. Especially, archaeal and eukaryotic genomes are good sources of tRNA introns that are removed by proteinaceous splicing machinery. Most intron-containing tRNA genes both in archaea and eukaryotes possess an intron at a so-called canonical position, one nucleotide 3′ to their anticodon, while recent bioinformatics have revealed unusual types of tRNA introns and their derivatives especially in archaeal genomes. Gain and loss of tRNA introns during various stages of evolution are obvious both in archaea and eukaryotes from analyses of comparative genomics. The splicing of tRNA molecules has been studied extensively from biochemical and cell biological points of view, and such analyses of eukaryotic systems provided interesting findings in the past years. Here, I summarize recent progresses in the analyses of tRNA introns and the splicing process, and try to clarify new and old questions to be solved in the next stages.

Progress in bioinformatics widens our understanding of structural characteristics of tRNA genes (Lowe and Eddy, 1997; Sugahara et al., 2006, 2008; Heinemann et al., 2010; Cognat et al., 2013). Especially, recent powerful sequence analyses with the next generation sequencers accumulate an enormous amount of sequence information in tRNA genes through whole genome sequencing of non-model organisms from various evolutional clades and through metagenome analyses mostly of prokaryotic species. In these analyses, introns were found in many tRNA genes in genomes among all of the three kingdoms of life (Heinemann et al., 2010). In eubacterial genomes, and their relatives, eukaryotic organellar genomes, small numbers of tRNA genes harbor the group I intron within the anticodon region (Reinhold-Hurek and Shub, 1992; Haugen et al., 2005). These introns are spliced by a series of phosphoester transfer reactions catalyzed by intronic sequences, whose mechanism is somehow related to splicing of mRNA. On the other hand, archaeal and eukaryotic nuclear genomes have tRNA introns whose splicing is completely dependent on proteinaceous enzymes (Phizicky and Hopper, 2010; Popow et al., 2012; Hopper, 2013). In addition to normal introns, their variations have been found in both archaeal and eukaryotic genomes. Furthermore, various interesting observations have been reported on biochemical and cell biological aspects in pre-tRNA splicing machinery in recent years. In this review, I mainly handle issues related to these “protein-spliced” tRNA introns and their splicing machinery by emphasizing comparison between archaeal and eukaryotic systems.

## Structural characteristics of tRNA genes harboring introns

**Figure 1:**
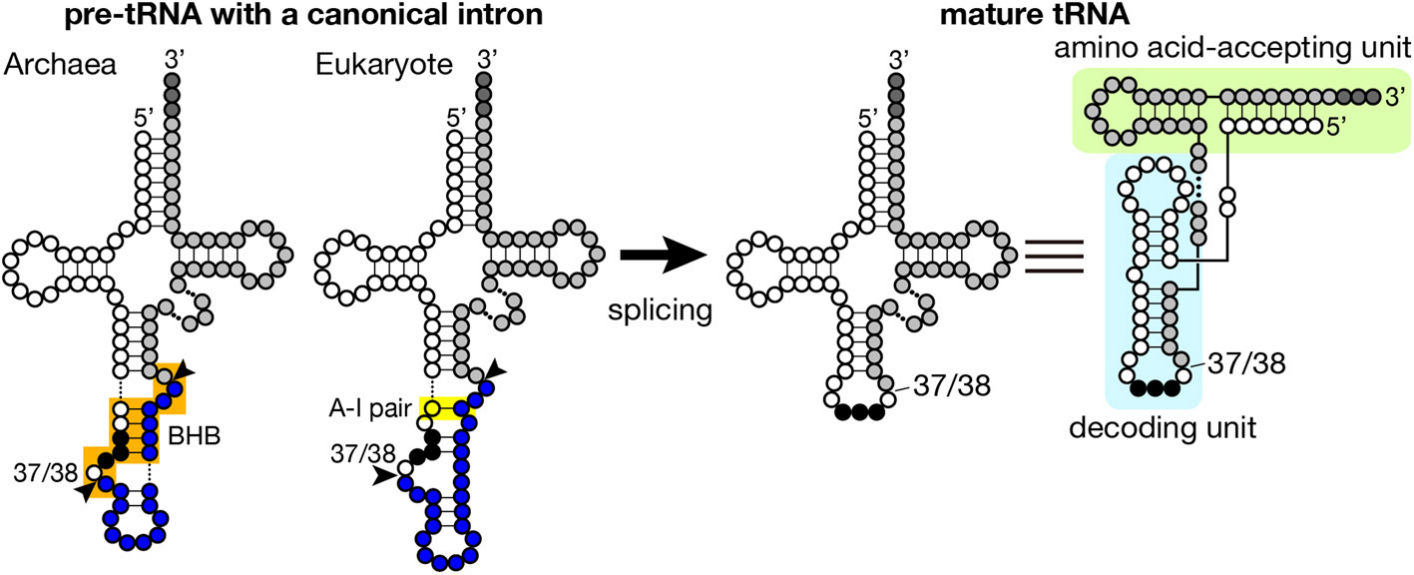
**Secondary structures of pre-tRNA and mature tRNA**. Typical secondary structures of pre-tRNAs with a canonical intron (left) and mature tRNA (right) are schematically shown. White circles with three black circles, blues circles, and light gray circles represent 5′-exon, intron and 3′ exon of pre-tRNA, respectively. The anticodon is represented with black circles, and CCA tri-nucleotides added at the 3′-terminus of tRNA are depicted with dark gray circles. The BHB motif in archaeal pre-tRNA and the A-I pair in eukaryotic pre-tRNA are highlighted with orange and yellow shadow, respectively. Arrowheads represent splice sites. Mature tRNA is illustrated in two different ways. In the right most structure, decoding and amino acid-accepting units of mature tRNA are marked with light blue and light green shadow, respectively.

Introns found in archaeal and eukaryotic tRNA genes are mostly inserted at one nucleotide 3′ to the anticodon, namely position 37/38 in the standard nomenclature (Figure 1) while introns are also inserted into other parts of tRNA genes in minor cases (see below in detail). Because intron insertion at this canonical position disrupts the anticodon stem-loop structure, its splicing is indispensable for tRNA maturation. On the other hand, the canonical intron does not seem to interrupt the overall tRNA structure (Figure 1). Indeed, pre-tRNAs with the canonical intron were shown to maintain structures of the D and TΨ C arms, and the acceptor stem by chemical and enzymatic probing (Swerdlow and Guthrie, 1984; Lee and Knapp, 1985). Structural characteristics of archaeal and eukaryotic tRNA genes containing the canonical intron have some similarity: mostly, 5′- and 3′-splice sites are set in short single-stranded segments franked by double-stranded stretches. However, close inspection of these structures reveal some difference between the two groups, which is derived from difference in strategy of splice site recognition by splicing enzymes. For splicing of pre-tRNAs, both archaebacteria and eukaryotes utilize similar sets of enzymes, namely tRNA splicing endonuclease (Sen) (Thompson and Daniel, 1990; Trotta et al., 1997; Li et al., 1998; Akama et al., 2000; Paushkin et al., 2004) and tRNA ligase (Phizicky et al., 1986; Englert and Beier, 2005; Englert et al., 2010, 2011; Popow et al., 2011, 2014). Some organisms require additional factors for the ligation step (Culver et al., 1997; Harding et al., 2008; Popow et al., 2011, 2014). Among these, splicing endonuclease is responsible for recognition of splice sites, and acts as a decoding engine of tRNA-type splice sites on various transcripts (see below in detail).

### Intron-containing tRNA genes in archaebacteria

Introns are found in every isodecoder tRNA (tRNAs with the same anticodon) genes of sequenced archaeal genomes. On an average, ~15% of tRNA genes have introns in the archaeal genomes while ratio of intron-containing genes varies from ~8% in *Euryarchaeota* to ~48% in *Crenarchaeota* (Marck and Grosjean, 2003; Sugahara et al., 2008; Chan et al., 2011). Length of introns ranges from 11 to 129 nt, and its median for each isodecoder tRNA falls mostly in 12–25 nt except the case of tRNA-Trp_CCA_ introns (65 nt), which nest a box C/D small RNA (Omer et al., 2000; Clouet d'Orval et al., 2001; Singh et al., 2004). The hallmark of splice sites in archaeal pre-tRNAs is the bulge-helix-bulge (BHB) motif (Kjems and Garrett, 1988; Tang et al., 2002; Marck and Grosjean, 2003), and the BHB motif is a critical determinant for recognition by archaeal splicing endonuclease (Xue et al., 2006), which consists of a 4 bp double-stranded helix flanked with two 3 nt bulges (Figure 1, shadowed with orange). In a pre-tRNA, the first two nucleotides of the anticodon base-pair with the intron to form a part of the central 4 bp helix, and the two adjacent bulges provide the 5′- and 3′-splice sites. The 5′- and 3′-splice sites may exist as independent entities, such as hBH and HBh′ motifs, in which splice sites are flanked by two short helices, in certain archaeal species (see non-canonical splice sites in **Figure 3**) (Marck and Grosjean, 2003). This secondary structure requirement in the BHB motif restricts a part of the primary sequence of the intron while quite large variations in sequence are accepted in the other parts of the intron.

**Figure 2:**
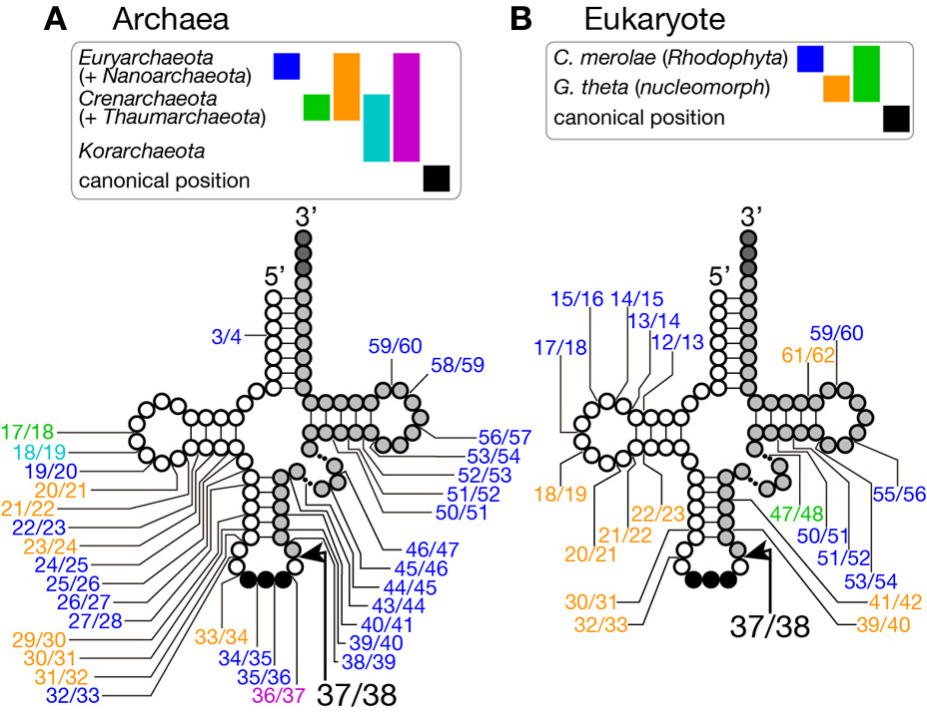
**Insertion points of non-canonical introns in tRNA genes**. Insertion points of non-canonical introns in various tRNA genes from archaea **(A)** and from eukaryotes **(B)** are shown. Positions of intron insertion found in certain groups of organisms are color-coded as shown on top of the tRNA models. Intron insertion points are summarized from data in Sugahara et al. (2008), Sugahara et al. (2009), and Chan et al. (2011) for archaea, and Kawach et al. (2005), Soma et al. (2007), and Maruyama et al. (2010) for eukaryotes.

**Figure 3:**
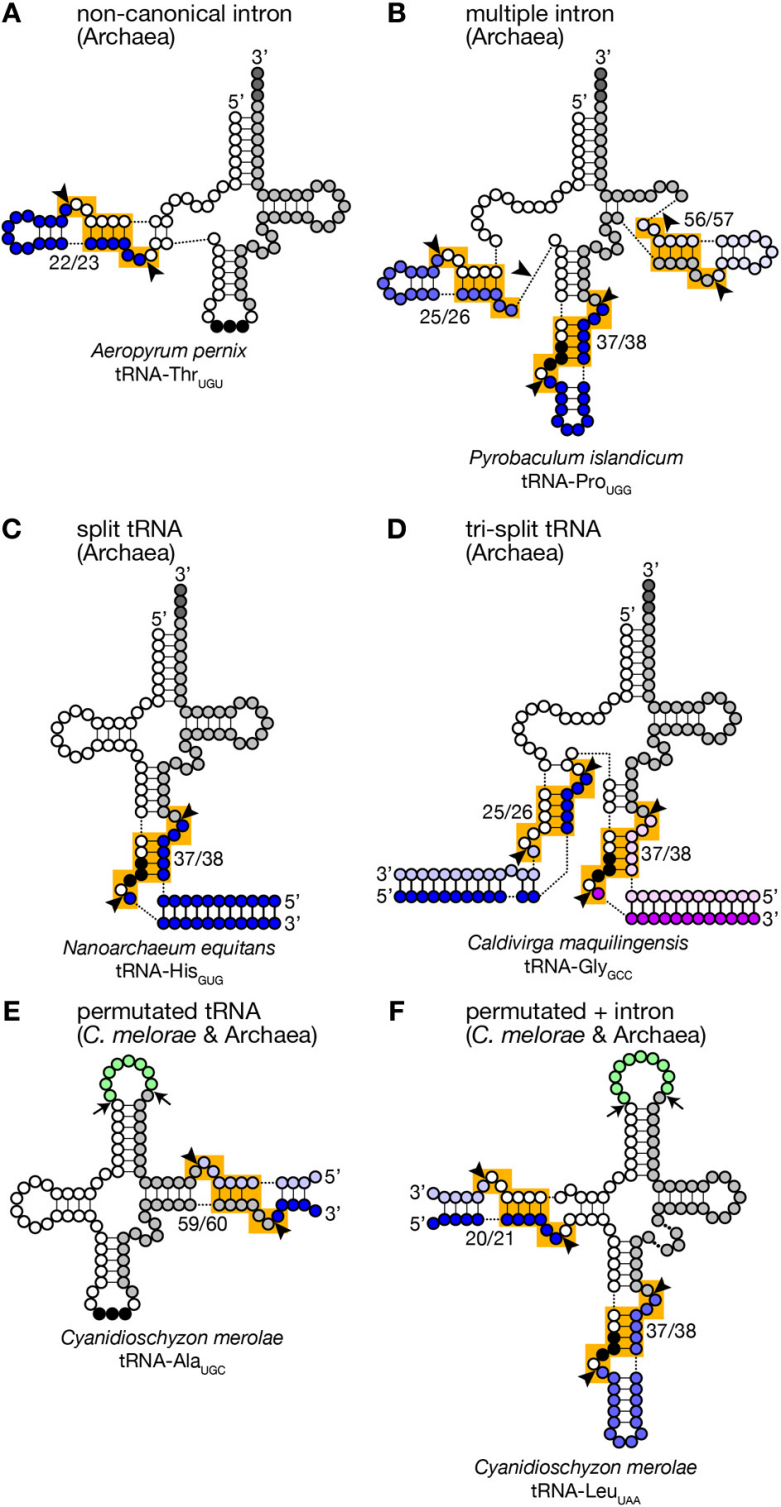
**Unusual tRNA genes**. Various examples of unusual tRNA genes are represented **(A–F)**. Exonic regions, BHB motifs and splice sites are marked as in Figure 1. Introns, leader and trailer sequences removed by splicing machinery are marked with bluish or purple-like colors. In panels **(E,F)**, linker regions between permutated tRNA fragments are colored in green, and cleavage sites are marked with arrows. tRNA secondary structures are drawn according to Chan et al. (2011), Sugahara et al. (2007), Randau et al. (2005a), Fujishima et al. (2009), and Soma et al. (2007).

Because of this transplantable nature of the splice sites, introns are also found at non-canonical positions of tRNAs, such as 20/21, 22/23, 25/26, 29/30, 30/31, 45/46, 53/54, 56/57, 59/60, etc., in some archaeal genomes (Figures 2A, 3). Especially, *Crenarchaeota* and *Nanoarchaeota* genomes are rich sources of non-canonical introns. In an extreme case, intron is inserted at position 3/4 in certain *Thermoproteales* tRNAs (Sugahara et al., 2008). The non-canonical intron is sometimes accompanied by the canonical and other non-canonical introns in the same tRNA genes. For example, a tRNA-Pro_UGG_ gene in *Pyrobaculum islandicum* has two non-canonical introns inserted at position 25/26 and 56/57 in addition to a canonical intron (Figure 3B) (Sugahara et al., 2007). Splice sites of these non-canonical introns are found not only in the BHB motif but also in the HBh′ and/or h′BH motifs (Marck and Grosjean, 2003).

Furthermore, tRNA genes consist of separated transcriptional units have been identified in Crenarchaeal genomes (Randau et al., 2005b,c). “Split tRNA genes” were first reported in *Nanoarchaeum equitans*: in this organism, tRNA-His_GUG_ is encoded by two gene fragments corresponding to 5′- and 3′-halves separated at position 37/38 in the anticodon loop (Figure 3C). The 5′- and 3′-halves are transcribed from their own promoters with the trailer and leader sequences whose portions are complementary to each other. A predicted secondary structure of the hybridized fragments is highly similar to that of a pre-tRNA harboring a canonical intron with a relaxed BHB motif, like a pre-tRNA received a cleavage at a loop in the intron. Indeed, RT-PCR analysis revealed that the split tRNA fragments are transcribed, and the transcripts are joined to form mature and functional tRNAs *in vivo*, indicating that *trans*-splicing is operating in *N. equitans* (Randau et al., 2005b,c). Further *in vitro* analyses revealed that splicing endonuclease from *N. equitans* can recognize this “pre-tRNA” complex and cleave off the trailer and leader sequences at the precise positions, and the resulting RNA is suitable for ligation by tRNA ligase (Randau et al., 2005a; Tocchini-Valentini et al., 2005). An interesting case in this organism is that tRNA-Glu_CUC_ and tRNA-Glu_UUC_, isodecoder tRNAs for the same amino acid, are produced from two different 5′-halves and one common 3′-half by *trans*-splicing (Randau et al., 2005b). Probably, *trans*-splicing contributes to saving genomic space to be assigned to tRNAs and to increasing probability to have more isodecoders especially in the case of *N. equitans*, a parasitic bacterium with massive genome reduction (Makarova and Koonin, 2005). Or *trans*-splicing may be an evolutionary remnant of the ancient form of tRNA gene organization (see below).

There are more complicated cases. In *Caldivirga maquilingensis*, tRNA-Gly isodecoders with anticodons, CCC, UCC, and GCC, are formed by combinations of up-to three out of five independent transcripts through *trans*-splicing (Figure 3D) (Fujishima et al., 2009; Sugahara et al., 2009). While tRNA-Gly_CCC_ is made from the 5′- and 3′-halves covering 1–37 and 38–73 regions, respectively, like the case of *N. equitans* tRNA-Glu_CUC_, the other two are joined from the 1–25 fragment common for the two, either one of the two specific fragments covering 26–37 with the anticodons, and the 3′-half used for all of the three tRNA-Gly isodecoders. Most of splits in the separated tRNA genes are located at canonical position (37/38) while those are at position 29/30 of tRNA-Ala_CGC_ and tRNA-Ala_UGC_, and at 25/26 of tRNA-Glu_UUC_ in the *C. maquilingensis* genome. *N. equitans* and some *Staphylothermus* genomes harbor split tRNA-Lys_CUU_ at position 30/31 (Fujishima et al., 2009; Chan et al., 2011). For the tri-split tRNA genes, splits are usually combination between one canonical position and one or more non-canonical positions (Fujishima et al., 2009).

### Intron-containing tRNA genes in eukaryotes

In eukaryotic genomes, most of intron-containing tRNA genes have their introns at the canonical position with length of 6–133 nt (Lowe and Chan, 2011). Because the nuclear genome of a eukaryote contains multiple tRNA genes encoding an isodecoder tRNA to allow sufficient supply of the tRNA, the number of tRNA genes in a eukaryotic genome does not correspond to that of sequence variations of tRNAs. Ratios of intron-containing tRNA genes vary significantly among eukaryotes: only 39 out of 1068 tRNAs genes (3.7%) have an canonical intron in *Strongylocentrotus purpuratus* (sea urchin) while in the infectious yeast, *Cryptococcus neoformans*, 132 out of 143 tRNA genes (92%) have an intron (Lowe and Chan, 2011). Whether all the predicted introns in eukaryotic genomes are spliced properly has not been fully confirmed. However, the yeast, *Saccharomyces cerevisiae*, which has 61 intron-containing genes encoding 10 different isodecoder tRNAs, can splice all the pre-tRNAs with introns by the single splicing endonuclease, the Sen complex, and this is also true in human (Trotta et al., 1997; Paushkin et al., 2004).

A clear difference between eukaryotic and archaeal introns in tRNA genes is that eukaryotic introns do not have clear local motifs specifying splice sites as described on archaeal introns (Figure 1). Rather, eukaryotic splicing endonuclease is considered to recognize splicing sites of pre-tRNAs by ruler-mechanism, in which the Sen complex measures distance between the body of tRNA and the splice sites, and this allows more flexible sequence selection around splicing sites in eukaryotic pre-tRNAs (Greer et al., 1987; Reyes and Abelson, 1988). Analyses of the secondary and tertiary structures of eukaryotic pre-tRNAs indicate that many pre-tRNAs have splice sites in single-stranded regions adjacent to double-stranded stretches, suggesting a eukaryotic site-recognition strategy similar to that of archaeal enzyme to some extent (Greer et al., 1987; Reyes and Abelson, 1988). Indeed, eukaryotic splicing endonuclease can recognize and splice archaeal-type pre-tRNAs with a BHB motif, meaning that eukaryotic splicing endonuclease has ability to read local structural features of substrate RNAs (Fabbri et al., 1998; Di Segni et al., 2005). However, close inspection of substrate specificity in tRNA splicing of *S. cerevisiae* and *Xenopus* extracts with artificial substrates demonstrated that double-stranded regions formed between the anticodon loop and the intronic sequence are not prerequisite to splicing (Reyes and Abelson, 1988). Thus, for our complete understanding of splice site properties essential for Sen recognition, we need to wait until structures of complexes between eukaryotic splicing endonuclease and various pre-tRNAs are solved.

Although short insertions can be found in D or TΨ C arms of various eukaryotic tRNA genes, such insertions are mostly found one of many synonymous tRNA genes on a genome. Thus, such genes are supposed to be pseudogenes, and non-canonical introns in eukaryotic genomes are supposed to be rare. However, it was reported that nuclear remnants of enslaved algae, nucleomorphs, in cryptophytes and chlorarachniophytes harbor tRNA genes with non-canonical introns in the D and TΨ C arms in addition to non-canonical positions in the anticodon stem-loop (Kawach et al., 2005; Maruyama et al., 2010). For example, the nucleomorph genome of *Guillardia theta* contains 9 tRNA genes (including one pseudogene) with insertions with length from 3 to 24 nt at non-canonical positions (Figure 2B). Interestingly, their excision, including that of the 3 nt intron in the TΨ C arm of tRNA-Cys_GCA_, tRNA-Val_AAC_, and tRNA-Leu_UAA(pseudo)_ genes, was confirmed by sequencing of RT-PCR products of the algal RNAs (Kawach et al., 2005) while we still do not know how these non-canonical introns, especially extremely short ones, are spliced. Moreover, 4 tRNA genes, such as tRNA-Phe_GAA_ genes, of this organism harbor two introns, and 2 tRNA genes, elongator tRNA-Met (tRNA-eMet) and tRNA-Cys_GCA_, do three introns, like the case of Crenarchaeal tRNA genes. Similar non-canonical introns are also found in the nuclear genome of a red algae *Cyanidioschyzon merolae* (Soma et al., 2007). Therefore, certain eukaryotic splicing endonucleases have ability to recognize splice sites inserted at non-canonical positions. In this point of view, splicing machinery of *C. merolae* seems to have more archaea-like characteristics, and BHB motifs seem to be critical determinants of substrate selection for this splicing endonuclease (Soma et al., 2013).

Another unusual tRNAs that were first reported on the *C. merolae* genome are permutated tRNAs, in which normal 5′- and 3′-termini are bridged with a short loop, and their 3′-half is positioned upstream of the 5′-half (Soma et al., 2007). Six tRNAs such as tRNA-Gln_CUG_ in *C. merolae* have its 5′-leader sequence before position 38 and its 3′-trailer after position 37. Atypical 5′- and 3′-termini are also introduced in the D arm of tRNA-Leu_UAA_, and the TΨ C arm in 4 tRNAs including tRNA-Ala_UGC_ (Figures 3E,F). RT-PCR analyses revealed that these permutated tRNA transcripts are indeed converted into mature and functional tRNAs via circular intermediates (Soma et al., 2007, 2013). The new 3′-trailer and 5′-leader sequences have ability to base-pair and produce a splice site-like structure with a BHB motif similar to that of archaeal split tRNA transcripts (Randau et al., 2005b). Thus, splicing machinery is thought to cleave the extensions from the permutated pre-tRNAs and joined separated ends of revers-oriented exons while true 5′- and 3′-termini of the mature tRNAs are thought to be generated by the action of terminal processing enzymes, such as RNase P and tRNase Z. Similar permutated tRNA genes have been found in other single-cell algae, such as prasinophytes and nucleomorphs in cryptophytes (Maruyama et al., 2010). Permutated tRNA genes are not limited to eukaryotes. An archaea, *Thermofilum pendens*, also has permutated tRNA-Tyr_GUA_ and initiator tRNA-Met (tRNA-iMet) genes with new 5′- and 3′-termini formed in the TΨ C arm (Chan et al., 2011). However, distribution of permutated tRNA genes is so far limited in only certain clades of eukaryotes and archaea.

## Splicing machinery

### Splicing endonuclease

As mentioned previously, splicing endonuclease is a search engine for tRNA-type splices sites among various transcripts produced by an organism. One splicing endonuclease, or an endonuclease complex, in an organism cleaves both the 5′- and 3′-splice sites of an intron to produce two 5′-OH termini on the intron and 3′-exon, and two 2′, 3′-cyclic phosphate termini on the 5′-exon and intron, meaning that the reaction is phosphoester transfer but not hydrolysis. The archaeal endonucleases are classified into three types from subunit organization; α_4_, α_2_ and α_2_β_2_ (Tocchini-Valentini et al., 2005; Calvin and Li, 2008). The archetypal configuration of the enzymes is supposed as an α_4_ homotetramer. The crystallographic analysis of EndA from *Methanococcus jannaschii* revealed that this tetrameric enzyme consists of a 2-fold but not 4-fold symmetric structure, where two subunits are mainly used to build two reaction centers for splice site cleavage, and the other two are used as structural components to position the catalytic subunits in an appropriate spatial arrangement for accepting the BHB motif (Lykke-Andersen and Garrett, 1997; Li et al., 1998). The α_2_ type is a kind of “a dimer of dimers,” in which two tandemly duplicated endonuclease units in a polypeptide act as a catalytic unit and a structural unit, respectively (Kleman-Leyer et al., 1997; Li and Abelson, 2000). The resulting dimer shows a configuration similar to that of α_4_ enzymes. The α_2_β_2_ type is also a derivative of α_4_ (Mitchell et al., 2009; Yoshinari et al., 2009). Gene duplication and different requirements for catalytic and structural subunits have led sequence divergence of the two subunits. Appearance of certain subunit types is well correlated with phylogenetic relation of archaebacteria and, more importantly, with diversity of tRNA introns and splice sites (Tocchini-Valentini et al., 2005; Calvin and Li, 2008). Species in *Euryarchaeota* and *Korarchaeota* essentially have α_4_- or α_2_-type splicing endonucleases, while those in *Nanoarchaeota, Thaumarchaeota*, and *Crenarchaeota* have α_2_β_2_-type enzymes. Interestingly, the latter group of archaea is rich in tRNA genes with introns: most unusual tRNA genes, such as those with non-canonical introns, with multiple introns or with permutated configuration, are found in these organisms (Marck and Grosjean, 2003; Sugahara et al., 2008; Chan et al., 2011). Some of the splice sites in these tRNA genes comprise expanded formats of the BHB, such as hBH or HBh′ (Tocchini-Valentini et al., 2005). On the other hand, *Euryarchaeota*, mostly possessing α_4_- or α_2_-type enzymes, has low ratios of tRNA genes with introns (less than 10%), and these tRNA genes have only one intron with the BHB motif at the canonical position. Therefore, subunit organization of splicing endonuclease seems to be a determinant for the substrate spectrum of the enzyme; strict to the BHB motif at the canonical position in α_4_ and α_2_ enzymes, and lenient in α_2_β_2_ enzymes.

**Figure 4:**
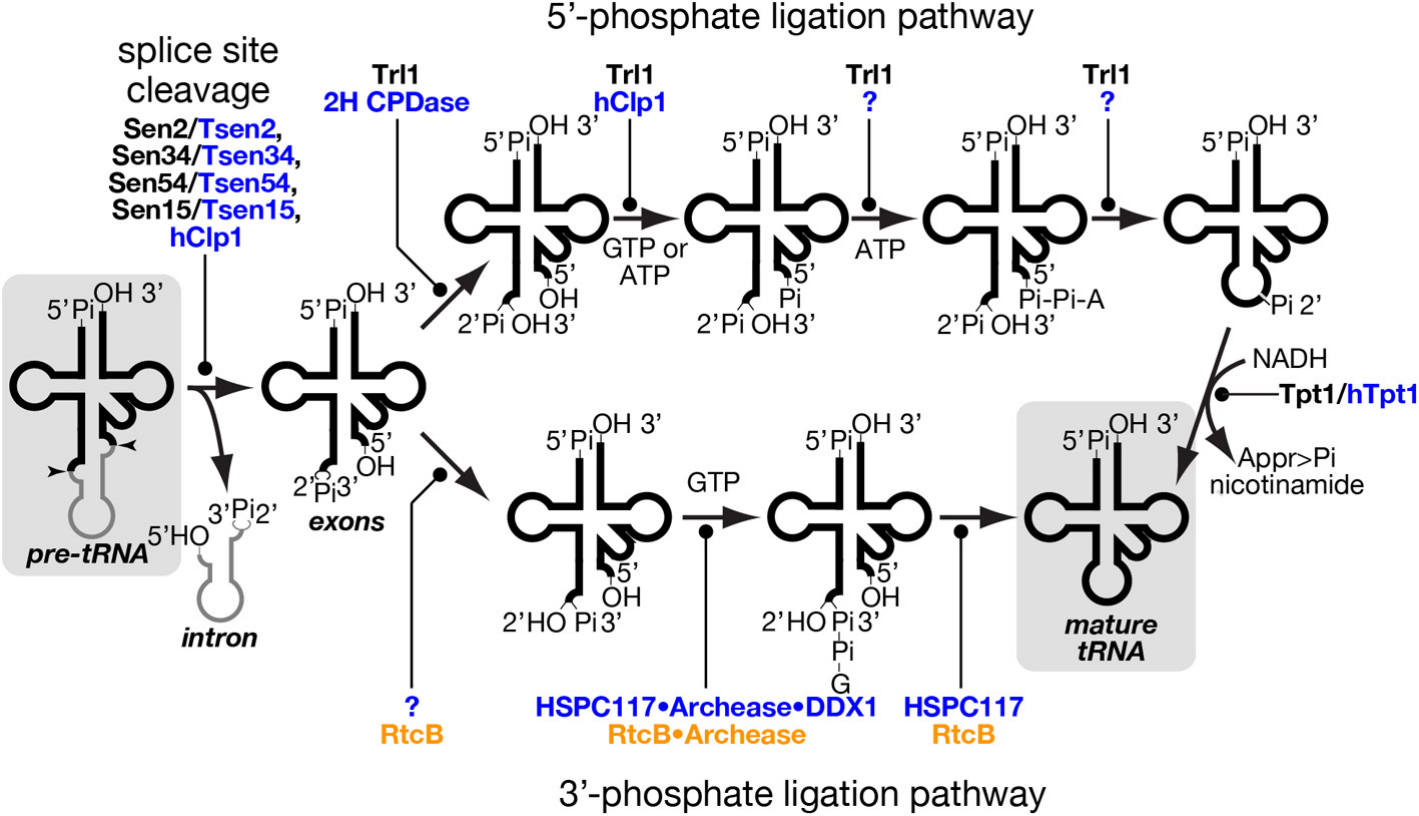
**tRNA splice site cleavage and two pathways of tRNA ligation**. Series of chemical reactions in splice site cleavage of pre-tRNA (left), the 5′-phosphate ligation pathway (upper), and the 3′-phosphate ligation pathway (lower) are shown schematically. Proteins involved in individual reactions are also shown, and unidentified factors are denoted by question marks. Those proteins of yeast, mammal and prokaryotes are color-coded with black, blue and orange, respectively. In the 5′-phosphate ligation pathway, yeast Tr11 uses GTP as a phosphate donor while hClp1 in the mammalian system uses ATP instead of GTP for 5′-phosphorylation of the 3′-exon. Although a mammalian enzyme(s) catalyzing the last two steps of this pathway is still missing, such an enzyme was found in lancelet. In the 3′-phosphate ligation pathway, the mammalian protein responsible for 2′-3′ cyclic phosphodiesterase in the HSPC117 complex was not fully identified, but eubacterial RtcB, an HSPC117 homolog, was demonstrated to possess this activity (Tanaka and Shuman, 2011). The starting material, pre-tRNA, and the end product, mature tRNA, are shadowed. Appr>Pi represents ADP-ribose 1″-2″ cyclic phosphate.

Eukaryotic splicing endonuclease consisting of four subunits, namely Sen2, Sen34, Sen54, and Sen15, was first identified in the yeast *S. cerevisiae* (Rauhut et al., 1990; Trotta et al., 1997). Among the four subunits, Sen2 and Sen34 have catalytic centers that are responsible for cleavage of 5′- and 3′-splice sites, respectively. On the other hand, Sen54 was reported to interact with the D-arm of substrate pre-tRNAs and to position catalytic subunits away from the body of tRNA with appropriate distances (Trotta et al., 2006; Xue et al., 2006). Human endonuclease contains homologs of all the 4 Sen proteins found in *S. cerevisiae* (Paushkin et al., 2004), and *Arabidopsis thaliana* also possesses Sen2 and Sen34 homologs (Akama et al., 2000). There is appreciable conservation among these eukaryotic Sen2 and Sen34 subunits, and archaeal endonucleases, indicating the same evolutional origin of splicing machinery. The human Sen complex also contains Clp1 (hClp1) (Paushkin et al., 2004; Weitzer and Martinez, 2007). Clp1 was first identified as a component of the cleavage factor II for polyadenylation of pre-mRNAs (de Vries et al., 2000), and then re-identified as an *in vitro* kinase for tRNA exons when searching for an enzyme phosphorylating siRNAs displaying a 5′-OH group (Figure 4) (Weitzer and Martinez, 2007). Divergence of subunit organization is greater in eukaryotic endonuclease than in archaeal enzymes, and eukaryotic splicing endonucleases have the ability to cleave both eukaryotic and archaeal types of splice sites as described above. Thus, it can be said that subunit organization diversity is, again, correlated with local splice site variability even between different kingdoms of life. Although direct relation between repertoires of intron-containing tRNA genes and subunit configuration of splicing endonucleases among eukaryotes has not been analyzed extensively, one interesting example is *C. merolae*. Bioinformatic analysis successfully identified genes for Sen2, Sen34, and Sen54 but failed to identify any gene or gene fragment that may encodes the fourth essential subunit Sen15 (Soma et al., 2013). In addition, yeast two-hybrid analyses could not identify other proteins that interact with either one of the three *C. merolae* Sen proteins, suggesting that the Sen complex of this organism may consist of 3 but not 4 subunits. Although further study is needed, such unique subunit organization in *C. merolae* may be related to existence of tRNA genes with non-canonical introns and permutated tRNA genes in this organism.

### Enzymes catalyzing ligation steps

Excised tRNA exons are joined by tRNA ligase. There are two completely different chemical pathways of ligation classified from the origin of the phosphate bridging the 5′- and 3′-exons: one is the 5′-phosphate ligation pathway and the other is the 3^′^-phosphate ligation pathway (Figure 4) (Popow et al., 2012). At the first step, ligation systems need to open the 2′, 3′-cyclic phosphodiester on the 5′-exon. The 5′-phosphate ligation pathway produces the 3′ terminus with 2′-phosphate and 3′-OH (Phizicky et al., 1986; Sawaya et al., 2003; Wang and Shuman, 2005) while the 3′-phosphate ligation pathway does that with 2^′^-OH and 3′-phosphate (Popow et al., 2011; Chakravarty et al., 2012). The former does not use the 2′-phosphate but a new phosphate derived from a nucleotide triphosphate to form a bridge between the two exons (Westaway et al., 1993), while the latter utilizes the 3′-phosphate left on the 5′-exon (Popow et al., 2011; Chakravarty et al., 2012). In both cases, the phosphorylated exon is further activated by nucleotidylation, and then ligated to its counterpart using the nucleotide monophosphate as a leaving group (Phizicky et al., 1986; Westaway et al., 1993; Popow et al., 2011; Chakravarty et al., 2012). In the case of the 5′-phosphate ligation pathway, the 2′-phosphate left at the splice junction must be removed after the ligation (Culver et al., 1997; Harding et al., 2008).

#### 5^′^-phosphate ligase

The 5′-phosphate ligation pathway has been found widely in fungi and plants. It also operates in lancelet but is most probably absent in mammalian cells (Lappe-Siefke et al., 2003; Harding et al., 2008; Englert et al., 2010; Popow et al., 2011). tRNA ligase for 5′-phosphate ligation, Trl1/Rlg1, was first identified in *S. cerevisiae* and then in *A. thaliana* through biochemical analysis (Phizicky et al., 1986; Englert and Beier, 2005). Trl1 homologs are widely found in the genomes from fungi through diatoms to angiosperms but are absent in animals (Englert and Beier, 2005; Wang et al., 2006). Trl1 homologs catalyze all the chemical reactions of exon ligation except for removal of the 2^′^-phosphate at the splice junction, and consist of three domains covering three enzymatic activities; adenylate synthetase/RNA ligase (ASTase), polynucleotide kinase (PNKase), and 2′, 3′-cyclic phosphodiesterase (CPDase) in this order (Xu et al., 1990; Sawaya et al., 2003). CPDase opens the 2′, 3′-cyclic phosphate on the 3′-terminus of the 5′-exon, and PNKase phosphorylates the 5^′^-terminus of the 3′-exon with GTP. By transferring an AMP moiety to this 5′-terminus from ATP, ASTase activates this end and allows ligation between the two exons (Figure 4, upper). During this terminal activation, the AMP moiety is first covalently attached to the ASTase domain and then transferred to the substrate. Although splicing endonuclease is responsible for primary recognition of splice sites, yeast Trl1 has the ability to interact with introns in pre-tRNAs (Tanner et al., 1988) and with an intron-containing precursor of its non-tRNA substrate, *HAC1* mRNA acting as a key component for the unfolded protein response (Sidrauski et al., 1996; Mori et al., 2010). The 2′-phosphate left at the splice junction is finally removed by 2′-phosphotransferase, or Tpt1 in *S. cerevisiae* (Culver et al., 1997). This reaction is unique in that 2′-phosphate is transferred to NADH to yield ADP-ribose 1″-2″ cyclic phosphate and nicotinamide (Figure 4, right most part). Indeed, this enzyme is essential for yeast growth, indicating that the 2′-phosphate is toxic for protein translation.

Although the 5′-phosphate ligation activity was once demonstrated in the nuclear extract of HeLa cells (Zillmann et al., 1991), a vertebrate 5′-phosphate ligase has not yet been identified. A lancelet *Branchiostoma floridae* has a complete set of enzymes for this ligation pathway; adenylate synthetase/RNA ligase activities and the other two activities for the tRNA ligation are encoded by two independent genes (Englert et al., 2010). In vertebrates, several polypeptides in different complexes can catalyze some of the individual reaction steps responsible for a putative 5′-phosphate ligation. First, *in vitro* phosphorylation of the 5′-terminus of the 3′-exon can be done by hClp1 kinase associated with the human Sen complex (Weitzer and Martinez, 2007). Second, mammalian genomes harbor genes for 2H CPDase, which can open the 2′, 3′-cyclic phosphodiester bond at the 3′-terminus of the 5′-exon to produce 3′-OH and 2′-phosphate groups, and the CPDase domain of fungal and plant Trl1 is classified as this family (Popow et al., 2012). Third, mammals possess 2′-phosphotransferase activities for RNA substrates. However, both mouse 2H CPDase and Tpt1 (Cnp1 and Trpt1, respectively) are non-essential for viability (Lappe-Siefke et al., 2003; Harding et al., 2008). Especially, Trpt1-knock-out mice, which completely lacks 2′-phosphotransferase activity, were demonstrated not only to translate Tyr-rich proteins efficiently, which require appropriate splicing of the tRNA-Tyr_GUA_ intron but also to show no defects in splicing of *XBP1* mRNA (Harding et al., 2008), which undergoes tRNA-type unconventional splicing in the cytoplasm upon unfolded protein response, like yeast *HAC1* mRNA (Yoshida et al., 2001; Calfon et al., 2002). Therefore, even if vertebrates have the complete set of the enzymes for the 5′-phosphate ligation pathway, this pathway seems to contribute to only a small, if any, part of RNA splicing.

#### 3^′^-phosphate ligase

In vertebrates, a tRNA ligase activity utilizing the 2′, 3′-cyclic phosphate to bridge the tRNA exons has been known for decades, but the enzyme responsible for this reaction was found only a few years ago (Filipowicz and Shatkin, 1983; Laski et al., 1983; Popow et al., 2011). Indeed, the 3′-phosphate ligase HSPC117 is a main player for tRNA splicing in mammals, and HSPC117 homologs are found in vertebrates, lancelets, insects, protozoa, algae etc., but not in fungi or angiosperms, which possess Trl1 homologs (Popow et al., 2011, 2012 for a review). Although HSPC117 forms a large complex with DDX1 (RNA helicase), CGI-99, FAM98B, and ASW, whether all of these subunits are involved in tRNA ligation is not known completely (see below). Homologs of HSPC117, whose name in prokaryotes is RtcB, exist widely in archaeal genomes where no Trl1 homologs exist (Englert et al., 2011). Interestingly, RtcB genes are also found in eubacterial genomes despite the fact that eubacteria do not have tRNA genes with introns removed by protein-assisted splicing. And eubacterial RtcB genes are located in operons with genes encoding RNA 3′-phosphate cyclase, which transforms a 3′-phosphate of an RNA molecule into a 2′, 3′-cyclic phosphate (Tanaka and Shuman, 2011). It is suggested that the set of enzymes is used to repair damaged tRNAs whose anticodon loop is endonucleolytically cleaved. These facts may indicate that the 3′-phosphate ligation pathway is a predominant and probably primordial pathway for tRNA ligation.

As mentioned above, HSPC117/RtcB also has adopted nucleotidylation to activate an exon terminus, but the ligase transfers a GMP moiety, instead of AMP, to the 3′-terminus of the 5′ exon (Figure 4, lower) (Chakravarty et al., 2012; Popow et al., 2014). During the reaction, the GMP moiety is covalently attached to the ligase, and this activation step seems to be rate limiting to the overall ligase cycle in eubacterial RtcB (Chakravarty et al., 2012). This is also true in human ligase HSPC117, and these facts suggest that some factor(s) is required for efficient turnover of 3′-phosphate ligase. Bioinformatic search for clusters of eukaryotic orthologous groups conserved in the same model organisms as RtcB and biochemical confirmation revealed that a small protein named “archease” enables HSPC117 to catalyze multiple-rounds of ligation reaction (Popow et al., 2014). Detailed biochemical analyses revealed that archease specifically enhances GMP transfer from GTP to HSPC117 protein but not that from the HSPC117-GMP adduct to the 3′-terminus of RNA substrates. Furthermore, this step also requires another activity catalyzed by DDX1, an RNA helicase within the HSPC117 complex. The implication of DDX1 ATPase provides evidence for molecular mechanism of ATP hydrolysis in the mammalian ligation reaction (Popow et al., 2011, 2014). Interestingly, involvement of archease in tRNA processing was first reported on m^5^C formation of tRNA in archaea *Pyrococcus abyssi*, where archease enhances substrate specificity of m^5^C methyltransferase and prevents it from aggregation probably by acting as a chaperone (Auxilien et al., 2007). Recently, archease was also demonstrated to catalyze efficient activation of archaeal RtcB where it enhances all nucleotidyl transfer steps in the 3′-phosphate ligation, including GMP transfer to RNA molecules (Desai et al., 2014).

### Diversity of splicing machinery in eukaryotes; intracellular localization and function

As mentioned above, eukaryotic splicing factors are more complex than those of archaea. According to this complexity, the splicing factors in various eukaryotes have acquired diversity in several aspects. One interesting aspect is the place of splicing in eukaryotic cells. Despite the functional and structural conservation of eukaryotic splicing endonucleases, their intracellular localization is divergent among organisms. Splicing endonucleases in vertebrates show expected localization: they are mainly found in the nucleus (De Robertis et al., 1981; Paushkin et al., 2004). Thus, it has been suggested that pre-tRNAs are spliced in the nucleus and only matured tRNAs are supplied to the cytoplasm. On the other hand, in *S. cerevisiae*, splicing of pre-tRNAs proceeds in the cytoplasm: tRNA splicing endonuclease was found to associate with mitochondrial surface, and this association is required for proper function of the enzyme (Yoshihisa et al., 2003, 2007). Later, the other enzymes required for completing splicing were also demonstrated to localize in the cytoplasm (Mori et al., 2010). It is to be noted that cytoplasmic localization of the Sen complex itself is not essential for pre-tRNA splicing for the yeast. When all the Sen subunits are expressed in the nucleus, pre-tRNAs are spliced normally (Dhungel and Hopper, 2012). These findings suggest that eukaryotic cells can tolerate drastic alteration of the place of tRNA splicing.

Although we have not obtained a complete view of tRNA splicing machinery in plant cells, some subunits of Sen proteins and tRNA ligase from *A. thaliana* were revealed to have multiple destination signals (Englert et al., 2007). When expressed as GFP fusions, AtSen2 and AtSen1 localized to the nucleus and mitochondria while AtTrl1 did to the nucleus and chloroplasts. Especially in the case of AtTrl1, alternative translational initiation may produce two isoforms; one from the most up-stream AUG is targeted to chloroplasts and the other from downstream AUG is to the nucleus (Englert et al., 2007). Thus, the nucleus may be the primary site of pre-tRNA splicing of nuclear encoded tRNAs in plant cells. On the other hand, one report provided a piece of evidence against this notion: unspliced pre-tRNAs accumulated when tRNA export from the nucleus was suppressed by RNAi-mediated knock-down of the tRNA export carrier, Exportin-t/Paused (Park et al., 2005). This situation is quite similar to that of *S. cerevisiae*, where disruption of *LOS1*, which encodes the yeast homolog of Exportin-t, leads accumulation of pre-tRNAs because of their sequestration from cytoplasmic splicing enzymes (Sharma et al., 1996; Yoshihisa et al., 2003). What is the role of mitochondrial or chloroplastic splicing enzymes in plants? Since there are no tRNA genes harboring eukaryote-archaea type introns in mitochondria and chloroplasts, plant splicing enzymes in these organelles should process a different type(s) of RNAs.

Evolution of eukaryotes also has hooked extra functions up to splicing machinery. As mentioned above, the yeast Sen complex can be transplanted from mitochondria to the nucleus without major defects in tRNA splicing. However, partial deletion of the mitochondrial targeting signal in Sen54 leads temperature sensitivity in yeast growth, and transplantation of the whole Sen complex to the nucleus compromises growth and rRNA maturation (Yoshihisa et al., 2003; Dhungel and Hopper, 2012). Thus, the yeast splicing endonuclease should have another essential function in the cytoplasm. Human splicing endonuclease activity also may have an extra function(s) other than pre-tRNA splicing. Mutations in human *SEN2, SEN34*, and *SEN54* (*TSEN2, TSEN34*, and *TSEN54* respectively) lead to pontocerebellar hypoplasia, which causes specific neurodegenerative disorders including hypoplasia of the cerebellum and the ventral pons, despite the fact that tRNA splicing is essential for every cell in the human body (Budde et al., 2008; Namavar et al., 2011). Similar phenotypes were observed when a *SEN54* homolog was knocked down in zebrafish (Kasher et al., 2011). Interestingly, this disease is also caused by mutations of mitochondrial arginyl-tRNA synthetase (Edvardson et al., 2007; Namavar et al., 2011). Although targets of human Tsen in the pontocerebellar cells related to this disease have not been identified so far, these facts suggest that Tsen has some neuronal cell specific function, other than usual tRNA splicing, through collaboration with mitochondrial arginyl-tRNA synthetase, another tRNA-related factor. Probably, because of their relaxed substrate recognition and expansion of repertoires of transcripts in eukaryotic cells, the Sen complexes from various eukaryotes may have adopted abilities to process and/or degrade various substrates in addition to intron-containing pre-tRNAs. Mutations in vertebrate Clp1 were also found to cause neurodegenerative phenotypes, such as motor-sensory defects, cortical dysgenesis and microcephaly, like the case of Tsen mutants (Hanada et al., 2013; Karaca et al., 2014; Schaffer et al., 2014). Mouse Clp1-K127A (kinase-dead) and hClp1-R140H (disease-related) compromise both pre-tRNA cleavage and 5′-RNA kinase activities *in vitro* while they specifically affect neuronal cells *in vivo* in mouse and human, respectively (Hanada et al., 2013; Karaca et al., 2014). Similar phenotypes were seen in *CLP1* mutants of zebrafish (Schaffer et al., 2014). The above facts suggest that, *in vivo*, residual activities of these Clp1 mutant proteins cannot complete tasks required in the neuronal cells while they can provide enough tRNA splicing ability in other cell types. Although fundamental functions of mammalian Clp1, as a subunit of the Sen complex or as a component of Cleavage Factor II generating mRNA 3′-ends, are carried out in the nucleus, some reports predict an extra-function in the cytoplasm. As mentioned before, hClp1 also acts as a 5′-kinase for siRNAs (Weitzer and Martinez, 2007). Phosphorylation of artificial siRNAs supplied from the outside of the cells may occur in the cytoplasm because only phosphorylated double stranded siRNAs are loaded to Ago proteins to yield RNA-induced silencing complexes in the cytoplasm while double stranded RNA import across the nuclear envelope has not been known (Nykänen et al., 2001; Ameres and Zamore, 2013). Indeed, Tsen-hClp1 can be purified from the cytosolic fraction (Weitzer and Martinez, 2007; Karaca et al., 2014).

High-order functions are also postulated for mammalian 3^′^-phosphate ligase. The HSPC117 complex was independently isolated as one of the components that allow axonal transport of mRNAs in neurons, suggesting that the HSPC117 has another function in the cytoplasm (Kanai et al., 2004). Indeed, the HSPC117 complex was recently found to shuttle between the nucleus and the cytoplasm (Pérez-González et al., 2014). A versatile RNA helicase DDX1 in this complex may account for such functions related to mRNA dynamics. As discussed above, both 5′-phosphate and 3′-phosphate tRNA ligases may also act as healing-sealing enzymes for damaged tRNAs.

## Physiological meanings to have an intron in a tRNA gene

### Physiological function of intron in pre-tRNA during its maturation

The tRNA intron is indeed an obstacle for tRNA's function, and seems to exist just to be removed after transcription. Why do organisms need to keep the intron on their tRNA genes? What are roles of the introns? So far, there are no clear answers to these questions. However, there are several examples that indicate some specific effects of tRNA introns on wide variety of aspects in the life of tRNAs and in the life of archaebacteria and eukaryotes.

**Figure 5:**
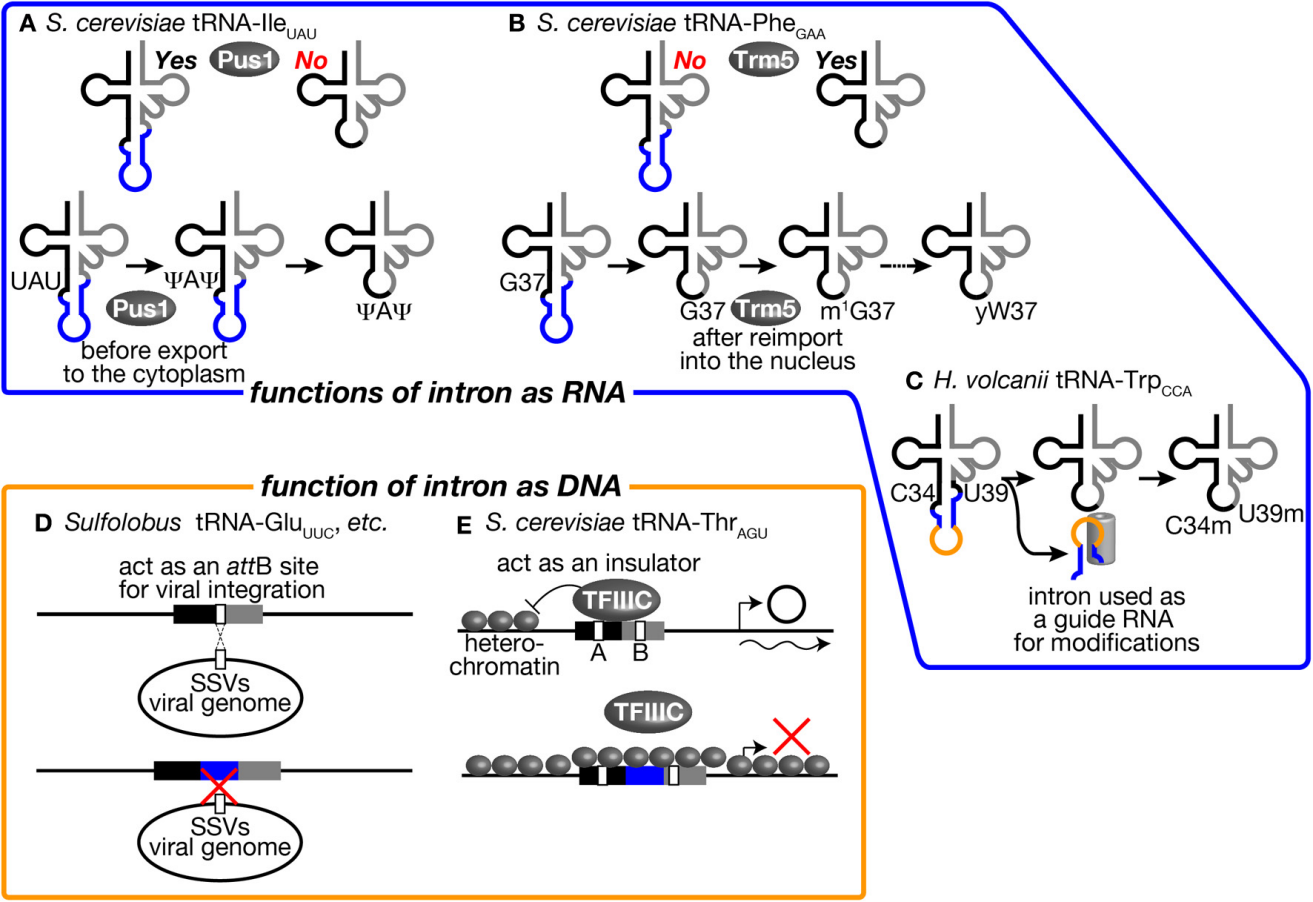
**tRNA introns affect various biological activities as RNA and DNA units**. Several examples where tRNA introns affect various biological activities as RNA units (upper) and as DNA units (lower) are illustrated. **(A)** The intron of tRNA-Ile_UAU_ in *S. cerevisiae* is an essential recognition motif for Pus1. Thus, pseudouridylation of U34 and U36 in the anticodon by Pus1 is only applied to the intron-containing pre-tRNA. Pre-tRNA-Ile_UAU_ pseudouridylated by Pus1 in the nucleus is subjected to cytoplasmic splicing after export from the nucleus in the yeast. **(B)** Yeast Trm5, a nuclear methyltransferase catalyzing the initial step of yW formation, only recognizes a spliced form of tRNA-Phe_GAA_, which is produced in the cytoplasm and re-imported into the nucleus. Thus, yW formation starts at a late stage of tRNA-Phe_GAA_ maturation. **(C)** The intron of tRNA-Trp_CCA_ in *H. volcanii* is used as a guide RNA to select two nucleotides, C34 and U39, of the same tRNA for 2′-*O*-methylation. **(D)** *Sulfolobus* SSVs use a part of tRNAs as an *attB* site for its integration into the host genome. Some *Sulfolobus* species harbor an intron in such tRNA genes to disrupt *attB* sequences. This may be a bacterial strategy to avoid viral infection. **(E)** tRNA-Thr_AGU_ near the *HMR*-E locus, which is in a heterochromatic region, acts as an insulator and prevents expansion of the heterochromatic region over the tRNA gene by recruiting TFIIIC properly in *S. cerevisiae*. The intron-containing tRNA-Leu_CAA_ gene cannot replace tRNA-Thr_AGU_ while the intronless version of the same tRNA can. See the text for details.

First, involvement of tRNA introns as recognition motifs for tRNA modification enzymes has been known in eukaryotes (Grosjean et al., 1997). Mainly from *in vitro* analyses, it was found that certain modifications in the anticodon loop are strictly applied on intron-containing pre-tRNAs. One famous example is that pseudouridylation of the 1st and 3rd U of the anticodon (U34 and U36) of tRNA-Ile_UAU_ in *S. cerevisiae* (Szweykowska-Kulinska et al., 1994; Simos et al., 1996; Motorin et al., 1998). tRNA-Ile_UAU_ needs to distinguish the AUA codon for Ile from AUG for Met. Conversion of UAU into Ψ AΨ is thought to enhance selectability to A against G at the wobble position. This pseudouridylation is applied only to intron-containing pre-tRNA-Ile_UAU_ by pseudouridine synthase Pus1 *in vitro* (Figure 5A, upper) (Simos et al., 1996; Motorin et al., 1998). In. *S. cerevisiae*, Pus1 localizes in the nucleus, and the splicing machinery does in the cytoplasm. Thus, the order of these processing events is guaranteed by the difference in intracellular localization (Figure 5A, lower). Interestingly, Pus1 also pseudouridylates at positions 25 and 67 of both spliced and unspliced forms of tRNA-Ile_UAU_. A similar example of intron-dependent modification is 2′-*O*-methylation of C34 in tRNA-Leu_CAA_ by Trm4 (Grosjean et al., 1997). Both yeast and human Trm4 methyltransferases require the intron in the substrate, tRNA-Leu_CAA_, *in vitro* (Strobel and Abelson, 1986; Brzezicha et al., 2006). The intron is also used to avoid premature modification during the series of modification reactions. tRNA-Phe_GAA_, derived from intron-containing genes, has an unusual nucleotide wybutosine (yW) at position 37 (Blobstein et al., 1975). The formation of yW starts with methylation of G37 by Trm5 in *S. cerevisiae*. Trm5 only recognizes the sliced but not intron-containing form of tRNA-Phe_GAA_ (Figure 5B, upper) (Noma et al., 2006). Again, the spliced form of tRNA-Phe_GAA_ first appears in the yeast cytoplasm because of the cytoplasmic localization of the splicing machinery. However, the spliced intermediate must be re-imported into the nucleus for this methylation since Trm5 localizes in the nucleus (Ohira and Suzuki, 2011). It has been shown that various tRNA species constantly shuttle between the cytoplasm and the nucleus, and the import system can deliver the spliced intermediate to the nucleus (Shaheen and Hopper, 2005; Takano et al., 2005; Shaheen et al., 2007; see also review, Hopper, 2013). After methylation by Trm5, tRNA-Phe_GAA_ with m^1^G37 is transported back to the cytoplasm to receive a series of chemical reactions to build yW at position 37 (Figure 5B, lower) (Ohira and Suzuki, 2011). In this case, the tRNA intron acts as a determinant of timing of modification.

In archaea, there is a unique example in which tRNA intron itself encodes a functional unit for modification of the body of the tRNA. In archaeal tRNA-Trp_CCA_ precursor, a box C/D small RNA is nested in the intron, and this intronic part of pre-tRNA-Trp_CCA_ or the excised intron is used to select nucleotides C34 and U39 for 2′-*O*-methylation *in trans* (Figure 5C) (Omer et al., 2000; Clouet d'Orval et al., 2001; Singh et al., 2004). Although these modifications in eukaryotes and archaea seem to be the driving force to maintain the introns in tRNA genes, the fact that Pus1 and Trm4 are dispensable for viability of *S. cerevisiae* is against this assumption (Simos et al., 1996; Motorin and Grosjean, 1999). It was also demonstrated that the intron of tRNA-Trp_CCA_ can be removed from the genome of *Haloferax volcanii*, suggesting that modification assisted by the intronic sequence itself does not cause strong selective pressure for intron maintenance (Joardar et al., 2008). Thus, no essential modifications only applied to intron-containing pre-tRNAs have been identified in eukaryotes and archaea so far.

### Possible functions of intron in tRNA genes on the chromosome

The intron may affect functions or abilities of tRNA genes on chromosomes. Although the primary function of tRNA genes is the source of genetic information to produce tRNAs, their well-structured and conserved sequences are utilized as targets of integration of viral genomes and other mobile genetic elements. Randau and Söll proposed that intron insertion will prevent tRNA genes from viral genome integration (Randau and Söll, 2008). Indeed, many archaeal viruses have been isolated from extreme environments, which tRNA-intron-rich archaea also prefer (Rice et al., 2001; Prangishvili, 2013). In well-studied cases in archaea, such viruses chose tRNA genes as integration sites. For example, *Sulfolobus* spindle-shaped viruses (SSVs) belonging to the *Fuselloviridae* family use either tRNA-Leu_GAG_, tRNA-Asp_GUC_, tRNA-Glu_UUC_, or tRNA-Glu_CUC_ as their *att*B sites, and they integrate their genome into the anticodon loop and TΨ C arm of these tRNA genes (Wiedenheft et al., 2004). By possessing an intron at or near the canonical position, or dividing tRNA genes into halves, such tRNA genes may escape from integration of these viruses (Figure 5D). However, there are other tRNA genes can be used as *att*B sites for these viruses, so that avoidance of viral infection may not be the sole reason to gain and/or maintain tRNA introns on archaeal genomes.

In eukaryotes, promoter elements are embedded in the coding region of tRNA genes, namely the A box and B box (Figure 5E) (Galli et al., 1981; Geiduschek and Kassavetis, 2001). These elements must be recognized by general transcription factor complexes in order for RNA polymerase III, especially TFIIIC, to initiate efficient transcription. Since the distance between the A and B boxes is altered by insertion of the intron, introns may affect recognition of the promoter elements by TFIIIC. Indeed, some early reports demonstrated that intron removal from reporter tRNA genes affects expression levels of some tRNAs in *Xenopus* oocytes *in vivo* and in yeast extracts *in vitro*, supporting the above assertion (Ciliberto et al., 1982; Fabrizio et al., 1987). On the other hand, other reports argue that yeast TFIIIC can bind to the tRNA promoter elements with various distance through its flexible linker between two recognition domains (Schultz et al., 1989; Camier et al., 1990). Recent genome-wide study of TFIII occupancy using ChIP/Seq analyses also revealed that rather even occupancy of Tfc1 (TFIIIC subunit) and Brf1 (TFIIIB subunit) on various tRNA genes (Nagarajavel et al., 2013). Therefore, alteration of distance between the A and B boxes by intron insertion *per se* may cause only minor effects on transcription of tRNA genes in general.

On the other hand, there is one example to indicate the effect of intron insertion on TFIIIC recognition of the tRNA promoter elements. It was reported that tRNA genes act as insulators or nucleosome phasing barriers (Donze et al., 1999). For this unique activity, the promoter elements of the tRNA genes must be properly occupied by TFIIIC (Simms et al., 2008). A tRNA-Thr_AGU_ gene, which does not have an intron, next to the yeast *HMR*-E locus acts as an insulator to block propagation of heterochromatin over this gene. On the other hand, a tRNA-Leu_CAA_ gene, which has a 19 nt-long intron, cannot act as an insulator, and removal of the intron brings insulator activity to the tRNA gene (Figure 5E) (Donze and Kamakaka, 2001). Therefore, possession of the intron can affect the additional function of tRNA genes related to genome organization.

### Direct examination of intron requirement

Essentiality of keeping introns in tRNA genes for organisms can be directly assessed by constructing strains without tRNA introns. As mentioned above, in archaea, one report demonstrated that the intron in the tRNA-Trp_CCA_ gene is dispensable for viability of *H. volcanii* in spite of its critical function in 2′-*O*-methylation of the tRNA (Joardar et al., 2008). By expanding similar analyses in certain archaea, it may be tested whether every tRNA gene harboring an intron(s) on an archaeal genome needs to possess the intron(s). On the other hand, the situation is not so simple in eukaryotes. As mentioned previously, most of isodecoder tRNAs are encoded by degenerated genes with the same or very similar sequences in eukaryotes. For example, even simple eukaryotes, such as yeasts, has more than 5 degenerated genes for each isodecoder tRNA on average. In *S. cerevisiae*, numbers of degenerated genes encoding intron-containing isodecoders varies from one for tRNA-Ser_CGA_ to 10 for tRNA-Leu_CAA_, tRNA-Phe_GAA_, and tRNA-Pro_UGG_ (Lowe and Chan, 2011). Thus, only one isodecoder, tRNA-Ser_CGA_, was examined for dispensability of its intron in 1980s despite versatility of the yeast in chromosomal modification through homologous recombination (Ho and Abelson, 1988). Indeed, it was found that the intron in the tRNA-Ser_CGA_ gene can be removed from the yeast chromosome without any apparent growth defects. However, the sequence of tRNA-Ser_CGA_ is quite similar to that of tRNA-Ser_UGA_; only three nucleotides are different between the two. Overproduction of tRNA-Ser_UGA_ can suppress lethality of deletion of the tRNA-Ser_CGA_ gene, and this suppression requires ncm^5^U modification at U34 of tRNA-Ser_UGA_ to expand its recognition repertoire at the wobble position (Johansson and Byström, 2004; Johansson et al., 2008). These facts suggest functional redundancy between tRNA-Ser_CGA_ and ncm^5^U-modified tRNA-Ser_UGA_ and that effects of intron removal from the tRNA-Ser_CGA_ gene may be masked by this redundancy. A stricter test was done recently with tRNA-Trp_CCA_, which is the only isodecoder to decode a single UGG codon for Trp and encoded by six genes with the same sequences by our group (Mori et al., 2011). Complete intron removal from all the tRNA-Trp_CCA_ genes conducted by repetitive replacement of the chromosomal tRNA genes with an intronless allele caused minimal effects on yeast growth under various conditions. In co-culture experiments with the wild-type strain up to 50 generations, the intronless strain even showed slight advantage over the wild type in growth. Furthermore, intron removal neither affects the amount and aminoacylation level of tRNA-Trp_CCA_, nor the protein synthesis rate while minor changes in protein composition were detected in 2D-PAGE analysis (Mori et al., 2011). Because there exists no other tRNA to decode Trp codons on the yeast genome, the above results indicate that the intron of tRNA-Trp_CCA_ is dispensable for viability of *S. cerevisiae*. Therefore, not strict but rather subtle effects to have introns in genes of certain isodecoder tRNAs may be advantageous for organisms during evolutional time scale under the natural conditions. Or, intron insertion to and intron loss from tRNA genes are rather neutral for organisms in evolution.

## Appearance of intron-containing tRNA genes and origin of introns

### Archaebacterial intron: establishment of archetypal intron-containing tRNA genes and modern intron-containing genes

There is a big debate on the origin of tRNA introns. One group argues for the “intron-first” scenario: all the primordial tRNA genes had an intervening sequence to be removed on the anticodon loop or consisted of a set of two halves, and then a large part of such introns have lost from the tRNA genes or the two halves have been joined on the genome during evolution (Di Giulio, 1992, 2006). The other argues for the intron-late scenario that introns were inserted after primordial tRNA genes had been established (Cavalier-Smith, 1991). From the “simple-to-complex” view, the hypothesis that tRNA is derived from a tandem duplication of a short stem-loop or ligation of two fragments may be reasonable. Indeed, existence of split tRNAs, especially those create multiple tRNAs by combination of 5′- and 3′-halves, fits to this idea (Di Giulio, 2006). Phylogenetic analysis of archaeal tRNA genes also suggests that 5′- and 3′-halves of tRNA genes even without introns have evolved through different evolutional trails (Fujishima et al., 2008). One and critical problem of this hypothesis is that functional units of a tRNA molecule, a decoding unit and an amino acid-accepting unit, do not correspond to these possible ancestral stem loops (Figure 1).

On the other hand, comparison of closely related species supports the idea that some “modern” introns have been inserted into tRNA genes afterwards. Many tRNA genes encoding different isodecoders in *Thermoproteales* genomes possess introns with very similar sequences at canonical and non-canonical positions despite the fact that the tRNA bodies are mapped at different phylogenetic positions (Fujishima et al., 2010). This finding suggests that large-scale intron transposition occurred in this order of archaea after the species had been established. Although the mechanism of intron transposition is still unknown, this is allowed because the BHB motif is required at exon-intron junctions and may be able to specify possible transposition targets on the chromosome. Thus, this type of intron gain by archaeal tRNA genes is supposed to proceed in the DNA level.

### Eukaryotic intron: possibility of “recent” gain of tRNA intron

**Figure 6:**
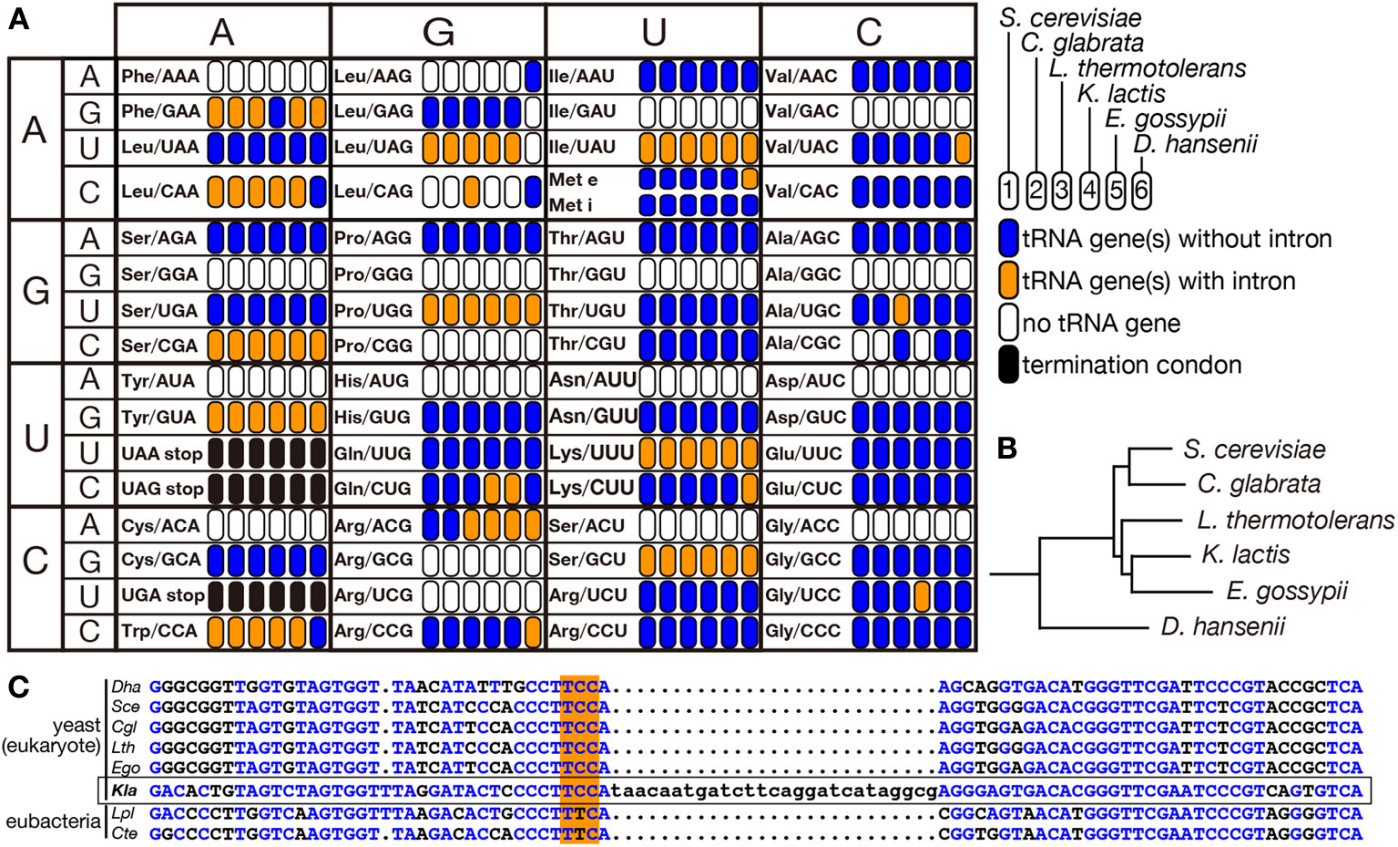
**Distribution of intron-containing tRNA genes in yeast genomes**. Distribution of intron-containing genes in 6 representative yeast genomes (*Saccharomyces cerevisiae, Candida glabrata, Lachancea thermotolerans, Kluyveromyces lactis, Eremothecium gossypii*, and *Debaryomyces hansenii*) is summarized in the anti-codon table **(A)**. As illustrated in the right, existence of intron-containing (orange) or intronless (blue) tRNA genes for each anticodon position is color-coded. Anticodon positions to which no tRNA genes are assigned on the genome or those correspond to termination codons are shown in white and black, respectively. The phylogenetic tree of these yeasts shown in **(B)** was drawn according to Génolevures Consortium (2009). Among these yeast, *D. hansenii* does not belong to *Saccharomycetaceae* and is used as an outgroup. **(C)** Sequence comparison of tRNA-Gly_UCC_ from *D. hansenii* (*Dha*), *S. cerevisiae* (*Sce*), *C. glabrata* (*Cgl*), *L. thermotolerans* (*Lth*), *E. gossypii* (*Ego*), and *K. lactis* (*Kla*), and tRNA-Glu_UUC_ from *Lactobacillus plantarum* (*Lpl*) and *Clostridium tetanus* (*Cte*). Nucleotides identical to those of *K. lactis* tRNA-Gly_UCC_ (marked with a frame) are shown in blue, and anticodons are highlighted with orange shadow.

Eukaryotes are thought to have evolved from an ancestral prokaryote more closely related to archaebacteria than eubacteria. So that, most of modern eukaryotic species might have lost archetypal intron-containing tRNA genes, which may be found in some of the archaeal genomes. Spectra of tRNA genes harboring an intron in eukaryotic genomes are not correlated with evolutional trails. Indeed, certain eukaryotic species possess intron-containing genes for a certain isodecoder tRNA while other species in the same clade do not. For example, the yeast *Lachancea thermotolerans* in *Saccharomycetaceae* has 3 intron-containing tRNA-Ala_UGC_ genes and 3 intron-containing tRNA-Leu_CAG_ genes while other *Saccharomycetaceae* yeasts have only intron-less genes for these isodecoders (Figure 6A). From the sequencing analysis, the intron-containing tRNA-Leu_CAG_ genes seem to have emerged through codon switching by U34C mutation from tRNA-Leu_UAG_, which is mostly encoded by intron-containing genes in the *Saccharomycetaceae* yeasts. Thus, unique appearance of an intron on genes for a certain isodecoder tRNA does not necessarily mean intron gain of the tRNA genes during evolution. On the other hand, there is no tRNA paralogs encoded by intron-containing genes for tRNA-Ala_UGC_ in the *Saccharomycetaceae* yeasts. Mature parts of this isodecoder from *L. thermotolerans* are highly homologous to those of the same isodecoder from other related yeasts. Thus, tRNA-Ala_UGC_ genes in *L. thermotolerans* probably acquired their intron during evolution. Another unique piece of evidence for gain of a modern intron by tRNA genes is tRNA-Gly_UCC_ in *Kluyveromyces lactis*. In *Saccharomycetaceae*, only *K. lactis* has two tRNA-Gly_UCC_ genes with a eukaryotic intron. Surprisingly, the sequence of mature tRNA-Gly_UCC_ from *K. lactis* is less homologous to those of the same isodecoder from other yeasts, including those from the most related genera such as *Lachancea* and *Eremothecium* (Figure 6B), but more homologous to tRNA-Glu_UUC_ from a eubacterium, *Lactobacillus plantarum*, where no protein-spliced intron exists in tRNA genes (Figure 6C). These situations strongly suggest that a bacterial tRNA-Glu_UUC_ gene was horizontally transferred to the genome of *K. lactis* or its close ancestor and was converted into tRNA-Gly_UCC_ by U35C mutation. Then, the tRNA-Gly_UCC_ gene seems to have acquired an intron. Thus, some, if not all, of introns in eukaryotic tRNA genes are suggested to have modern origins. There are many opposite cases, in which only one species lacks the intron of a certain isodecoder tRNA while the same isodecoder of other related species is encoded by intron-containing genes (see tRNA-Phe_GAA_ in Figure 6A), suggesting intron-loss from tRNA genes during evolution.

Although it is easy to postulate ways of intron-loss from tRNA genes during evolution, how can a eukaryotic tRNA gene gain such a modern intron at the canonical position? Not like the case of archaea, sequences of exon-intron junctions in eukaryotic pre-tRNAs do not show clear structural characteristics as mentioned above. Therefore, it is difficult to imagine mechanisms by which a DNA fragment corresponding to a tRNA intron is transposed to another position on a chromosome. Another possibility is that intron is inserted into a tRNA gene via a reverse-transcribed intermediate. It is to be noted that the anticodon loop, into which eukaryotic tRNA introns are mostly inserted, is often the target of tRNA cleavage enzymes. To recognize a codon on mRNAs, the anticodon loop must be accessible to macromolecules while the D- and TΨ C-loops interact with each other via base-pairing to form a tight structure. Thus, the anticodon loop is an Achilles' tendon for the tRNA to receive undesired cleavages. Well-known examples are so-called ribotoxins, such as colicin E5, and PrrC endonucleases produced by *Escherichia coli*, which cleave the anticodon of certain tRNAs to kill surrounding competitor bacteria and to kill itself when infected by a deadly pathogen, bacteriophage, respectively (Kaufmann, 2000). Similar anticodon nucleases are also found in eukaryotes. γ-Subunit of zymocin, produced by *K. lactis*, enters *S. cerevisiae* cells and cleaves tRNA-Glu_UUC_ if U34 of the anticodon is modified to mcm^5^s^2^U (Lu et al., 2005). Eukaryotic cells also have tRNA cleavage enzymes whose targets are tRNAs in their own cells, such as Rny1 in yeast and angiogenin in mammals (Thompson and Parker, 2009; Yamasaki et al., 2009). tRNA cleavage activities of these endonucleases are regulated by cellular stresses, and the resulting tRNA fragments are used to suppress translation (Ivanov et al., 2011; Luhtala and Parker, 2012). Interestingly, the cleavage catalyzed by zymocin cannot be healed by tRNA ligase of *S. cerevisiae*, while heterologous expression of *A. thaliana* tRNA ligase can rescue *S. cerevisiae* from the deleterious effect of zymocin (Nandakumar et al., 2008). Probably, cleavage of the anticodon loop by these endonucleases is carried out so as to escape emerged RNA termini from endogenous tRNA ligase. Thus, the termini may provide initiation sites from which RNA-dependent RNA polymerase extends the 5′-tRNA halves with unidentified templates, and then eventually its 3′-terminus may be ligated with the 5′-terminus of the 3′-half by tRNA ligase if these ends are recognized by healing-sealing activity of tRNA ligase (Schwer et al., 2004; Nandakumar et al., 2008). Even though such cleavage, elongation and healing might occur in the cytoplasm, the resulting tRNA derivatives might be re-imported into the nucleus using tRNA import machinery (Shaheen and Hopper, 2005; Takano et al., 2005). Thus, the tRNA derivatives can be subjected to reverse transcription and integration into the genome in the nucleus. Or, if the cleavage occurs in the nucleus, reverse transcriptase may directly elongate the 5′ fragment.

Both loss and gain of tRNA introns seem to have occurred during various stages of eukaryotic evolution. In addition to such events, eukaryotic genomes require another layer of gene arrangements to settle their tRNA gene repertoires. All the genes encoding a certain isodecoder tRNA on a eukaryotic genome tend to have an intron or they do not at all. Even in the cases that an isodecoder tRNA is encoded by a mixture of intron-containing and intron-less genes, those intron-containing and intron-less genes are supposed to have different origins. For example, human tRNA-Leu_CAA_ is encoded by total seven genes; one intron-containing gene on Chr I, four intron-containing genes on Chr VI, one intron-less gene on Chr I, and one intron-less gene on Chr XI. Sequence comparison revealed that the intron-less gene on Chr I is more related to tRNA-Met in dog and mouse than the human intron-containing tRNA-Leu_CAA_ genes while that on Chr XI is more related to tRNA-Leu_UAG_ in human and zebrafish. Therefore, sequences of tRNA genes with the same origin are supposed to be equalized including their intronic part. This is also confirmed among species. If sequences of tRNA-Trp_CCA_ genes are compared among fungal genomes, those from the same organism are clustered (Mori et al., 2011). Intergenic conversion between highly homologous tRNA genes for an isodecoder may contribute to this sequence equalization. Indeed, intergenic spreading of mutations among tRNA genes of the same isodecoder have been known for years as intergenic gene conversion hot spots (Amstutz et al., 1985; Heyer et al., 1986). Although tRNA genes are scattered around the whole yeast chromosomes, they are closely located around the nucleolus in *S. cerevisiae*, and near centromeres in *Schizosaccharomyces pombe*, which has only three chromosomes, and this intra-nuclear localization is driven by interaction between TFIIIC and condensin (Thompson et al., 2003; Haeusler et al., 2008; Iwasaki et al., 2010). Such spatial positioning of tRNA genes in the nucleus is supposed to help efficient equalization of tRNA sequences through intergenic gene conversion. We still do not know what determines balance between domination of intron-containing genes and that of intron-less genes. As described before, there seems to be no essential difference in function of tRNA molecules derived from intron-containing and intron-less genes. It might be possible that domination of intron-containing or intron-less tRNA genes for an isodecoder tRNA just comes from stochastic change between the two states during the evolution: if the one of the two types occupies all the tRNA loci, no change becomes possible until a new and very rare event of intron-loss or intron-gain occurs.

Here, I have gone through recent progresses related to tRNA introns found in both archaea and eukaryotes. The life of a tRNA molecule transcribed from intron-containing genes has been studied for decades from various points of views, and has provided various interesting findings in bioinformatics, in molecular biology, in cell biology, and even in human pathology. However, accumulating information from the experimental and computational analyses has brought us more questions to be solved, especially those related to eukaryotic tRNA introns and their splicing. Still, we do not understand completely how eukaryotic splicing endonuclease decodes hidden motifs of splice sites in eukaryotic pre-tRNAs. What kinds of substrates other than pre-tRNAs does splicing machinery process in archaea and eukaryotes? Why can tRNA introns be spliced in different intracellular compartments among eukaryotes? Are there any other physiological roles of tRNA introns in the life of tRNA or those as genetical elements on the chromosomes? What are real selective pressures to keep introns in tRNA genes if existing? Most importantly, what are origins of modern tRNA introns, and how have they appeared and disappeared in archaeal and eukaryotic genomes during evolution? Some of the questions may not be able to be answered easily, but our quest of the life of tRNAs to seek the answers is going on.

### Conflict of interest statement

The author declares that the research was conducted in the absence of any commercial or financial relationships that could be construed as a potential conflict of interest.
